# Cardiac intensive care unit: where we are in 2023

**DOI:** 10.3389/fcvm.2023.1201414

**Published:** 2023-11-24

**Authors:** Amine Bouchlarhem, Zakaria Bazid, Nabila Ismaili, Noha El Ouafi

**Affiliations:** ^1^ Faculty of Medicine and Pharmacy, Mohammed First University, Oujda, Morocco; ^2^ Department of Cardiology, Mohammed VI University Hospital, Mohammed First University, Oujda, Morocco; ^3^ Faculty of Medicine and Pharmacy, LAMCESM, Mohammed First University, Oujda, Morocco

**Keywords:** acute cardiovascular care, cardiac intensive care unit, coronary care unit, acute coronary syndrome, healthcare system

## Abstract

Cardiac intensive care has been a constantly evolving area of research and innovation since the beginning of the 21st century. The story began in 1961 with Desmond Julian's pioneering creation of a coronary intensive care unit to improve the prognosis of patients with myocardial infarction, considered the major cause of death in the world. These units have continued to progress over time, with the introduction of new therapeutic means such as fibrinolysis, invasive hemodynamic monitoring using the Swan-Ganz catheter, and mechanical circulatory assistance, with significant advances in percutaneous interventional coronary and structural procedures. Since acute cardiovascular disease is not limited to the management of acute coronary syndromes and includes other emergencies such as severe arrhythmias, acute heart failure, cardiogenic shock, high-risk pulmonary embolism, severe conduction disorders, and post-implantation monitoring of percutaneous valves, as well as other non-cardiac emergencies, such as septic shock, severe respiratory failure, severe renal failure and the management of cardiac arrest after resuscitation, the conversion of coronary intensive care units into cardiac intensive care units represented an important priority. Today, the cardiac intensive care units (CICU) concept is widely adopted by most healthcare systems, whatever the country's level of development. The main aim of these units remains to improve the overall morbidity and mortality of acute cardiovascular diseases, but also to manage other non-cardiac disorders, such as sepsis and respiratory failure. This diversity of tasks and responsibilities has enabled us to classify these CICUs according to several levels, depending on a variety of parameters, principally the level of care delivered, the staff assigned, the equipment and technologies available, the type of research projects carried out, and the type of connections and networking developed. The European Society of Cardiology (ESC) and the American College of Cardiology (ACC) have detailed this organization in guidelines published initially in 2005 and updated in 2018, with the aim of harmonizing the structure, organization, and care offered by the various CICUs. In this state-of-the-art report, we review the history of the CICUs from the creation of the very first unit in 1968 to the discussion of their current perspectives, with the main objective of knowing what the CICUs will have become by 2023.

## 1. Introduction

Cardiac critical care is an area of intense basic, translational, and clinical research (1). This began with the establishment of the first coronary intensive care units (CCUs) dedicated to the management of acute coronary syndrome (ACS) in the 1960s (2). The main objective of this period was to develop the means of myocardial revascularization, first with the arrival of thrombolytic therapy, then with the development of percutaneous coronary interventions, first with balloon, then with stenting, which revolutionized the management of acute coronary syndrome. Over time, it has been shown that acute cardiac intensive care is not only limited to ACS but to other cardiovascular emergencies, for which reason CCUs have evolved into what are now called cardiac intensive care units (CICUs) (3). This evolution has been accompanied by a change in the phenotype of patients admitted to CICUs, as ACS is no longer the leading cause of admission ahead of cardiogenic shock and acute heart failure which currently dominate the rate of admissions to modern CICUs (4). According to the World Health Organization (WHO) 2020 mortality analysis report, cardiovascular disease has remained the leading cause of death worldwide for the past 20 years. However, the number of deaths from heart disease has increased by more than 2 million since 2000, reaching nearly 9 million deaths in 2019. As a result, heart disease now accounts for 16% of total deaths from all causes, and given the high mortality rate and the complexity of managing cardiovascular emergencies, the phenotype of patients who generally have several associated comorbidities, and the translational nature of cardiovascular emergencies, the development of these units was a crucial necessity (5).

The results observed during the first half of the 20th century did not show any decrease in intra-hospital mortality in patients hospitalized for a myocardial infarction in a medical service not equipped with personnel trained in intensive care, and not equipped with telemetric monitoring despite the therapeutic means used during that period. It was not until 1961, and after the alarming mortality rates of up to 30% (6) in patients hospitalized with coronary occlusion, that Desmond Julian (2) created the very first unit dedicated specifically to the hospitalization of coronary patients and named it the “coronary intensive care unit (CCU)”. Julian's vision was to decrease the mortality rate and he saw that it was necessary to have trained intensive care personnel, cardiopulmonary resuscitation (CPR) available in the hospital unit, and telemetric monitoring for all patients. Soon after, this concept was adopted by several healthcare systems, and the benefits of these units were evident from the first year of operation, with a significant decrease in the mortality rate to 15% after 1 year of activity (7) and between 3% and 6% after 2 years of activity (6).

## 2. The coronary care unit (CCU) concept

Desmond Julian was the first to introduce the concept of a unit dedicated only to patients with acute coronary syndrome, with the aim of early detection and treatment of ventricular arrhythmias, the main cause of death in these patients (8). According to Julian, in order to reduce mortality in patients with ACS, we need

• Continuous electrocardiographic monitoring with an alarm system that detects arrhythmias.
• Access to early and effective cardiopulmonary resuscitation with external defibrillation.
• All heart attack patients must be admitted to the same unit, where medical and paramedical staff have specialized training in cardiological care and are equipped with drugs that act on the heart.
• The ability of trained nurses to initiate cardiopulmonary resuscitation in the absence of doctors is at the origin of the concept of the Coronary Intensive Care Unit (CCU).

**Figure 1:**
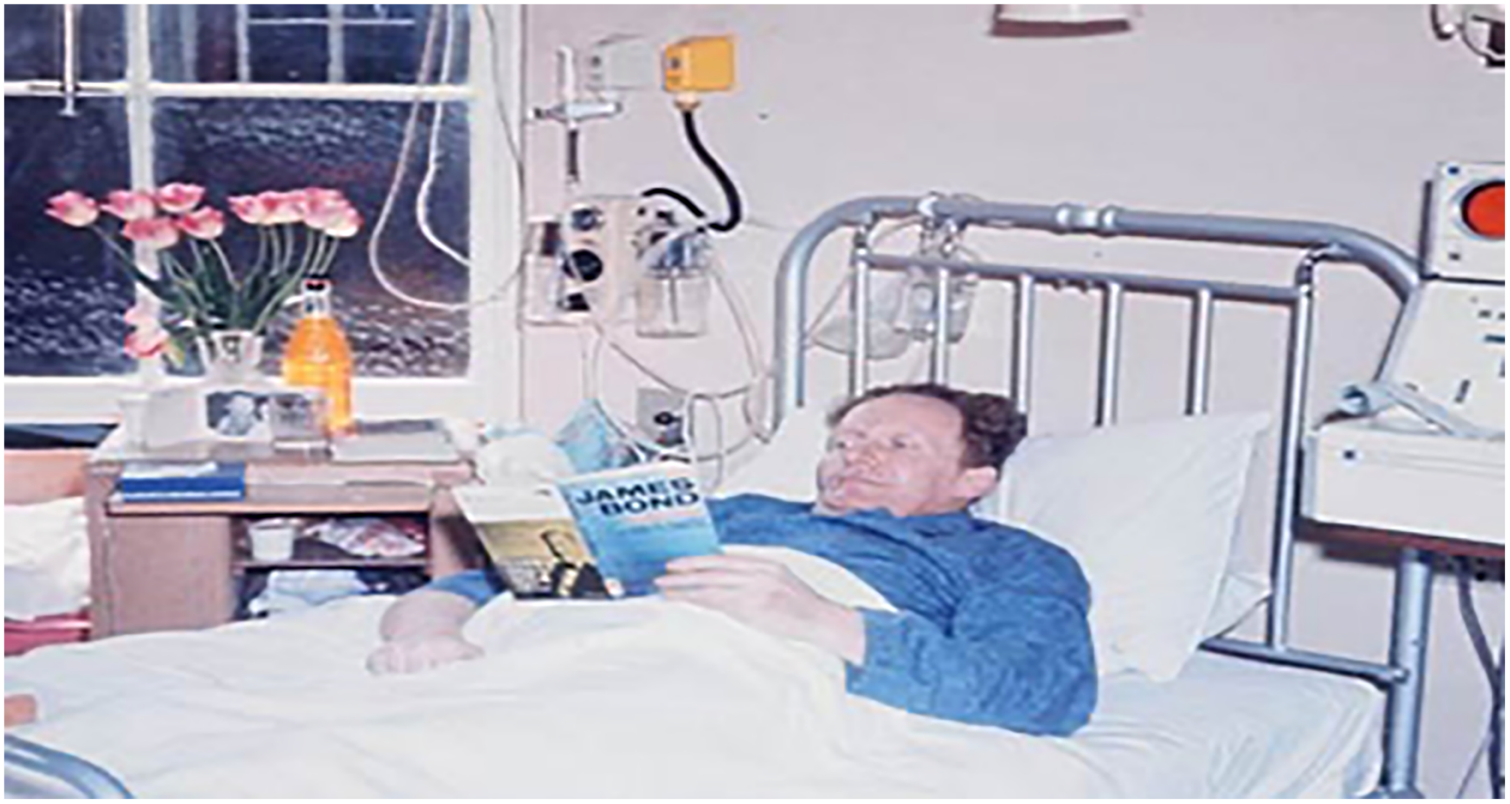
Desmond Julian simulates the experience of the first patient admitted to the coronary intensive care unit at Sydney hospital [reproduced with permission from (8)].

For these reasons, Desmond Julian founded the first coronary intensive care unit in Sydney in October 1961 (Figure 1) (8) and is considered the pioneer of the concept. The concept was rapidly adopted in Canada, with Kenneth Brown transforming a small four-bed room into a coronary intensive care unit at Toronto General Hospital (Canada) in March 1962, with Hughes Day adopting the concept at Bethany Hospital in Kinshasa in May 1962, and Lawrence Meltzer and Roderick Kitchell at Presbyterian Hospital in Philadelphia in November 1962 (9, 10) (Table 1).

**Table 1:** Summarizes the chronology of the founding of the first coronary intensive care units.

Hospital structure	Founder	Date
Sydney Hospital	Desmond Julian	October 1961
Toronto General Hospital	Kenneth Brown	March 1962
Bethany Hospital in Kinshasa	Hughes Day	May 1962
Presbyterian Hospital in Philadelphia	Lawrence Meltzer et Roderick Kitchell	November 1962

**Figure 2:**
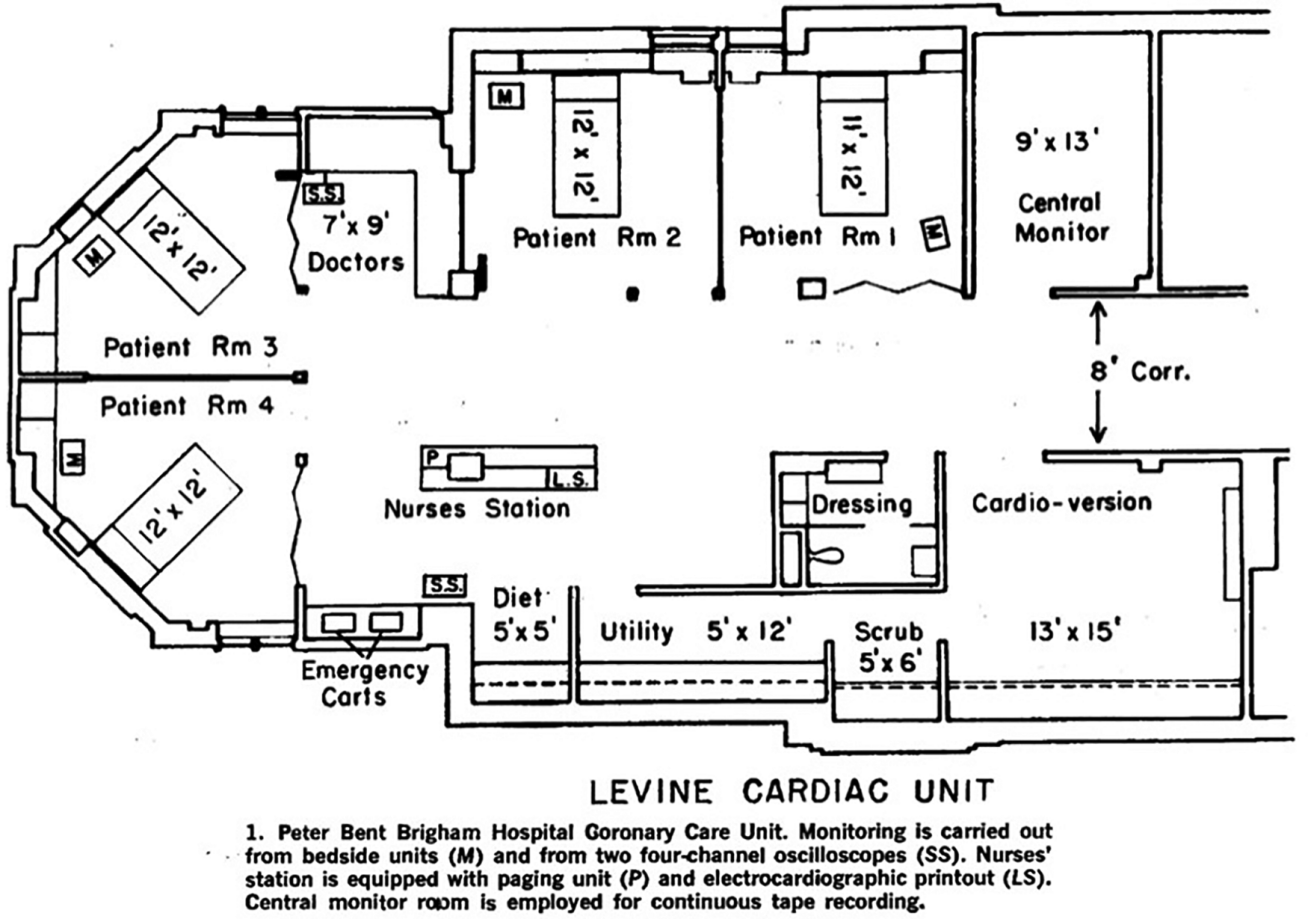
The architectural plan of the coronary intensive care unit founded by Bernard Lown at the peter bent brigham hospital (15).

In 1967, Bernard Lown published an article in the American Journal of Cardiology on the new perspectives and orientations of the CCU. Firstly, he presented the unit in which he practiced at the Peter Bent Brigham Hospital, a unit with four private single rooms, each equipped with a continuous electrocardiographic monitor, characterized by the presence of an alarm for arrhythmias and severe variations in heart rate. Adjacent to the rooms was a monitoring room dedicated to the nursing staff, equipped with a large oscilloscope showing all inpatient patterns (Figure 2) (11). According to Lown and colleagues, among the many rhythm changes in the acute phase of myocardial infarction, there are those that are benign and should be ignored and others that are more serious and should be considered prodromes of serious arrhythmias, mainly premature ventricular contraction (11, 12) bradycardia and finally atrioventricular block (AVB). The management of arrhythmias should include preventive treatment, as well as the removal of triggering factors, mainly pain, extreme bradycardia, heart failure, and psychological stress.

After the well-deserved success in preventing severe arrhythmias and decreasing the in-hospital mortality rate, the next battle was the problem of heart failure, since it was becoming the leading cause of death along with cardiogenic shock. Several studies were interested in studying the effects of myocardial infarction (MI) on the cardiorespiratory and hemodynamic systems, and despite the difference in study methods and patient phenotypes, there was a consensus on the hemodynamic and respiratory changes after MI, especially in patients with cardiogenic shock (13, 14). The most typical alteration was the association of a decrease in cardiac output associated with an increase in peripheral vascular resistance (16).

In 1970, one of the great advances in the evaluation of the cardiac pump in MI was the pulmonary artery catheterization used by Swan and Ganz, hence the name “Swan Ganz catheter” or “invasive hemodynamic monitoring by the Swan Ganz method” (17), which allowed the adaption of the medical treatment of heart failure in the acute phase of an MI according to the degree of failure by setting up a classification based on cardiac index, capillary pulmonary pressure, and clinical signs (18). This invasive hemodynamic monitoring, which has become routine in the daily practice of patients hospitalized in coronary intensive care units in North American countries, was little practiced or even neglected, in the United Kingdom, due to the limited number of centers with the expertise and resources necessary for this type of monitoring (2). Over time, invasive monitoring has become increasingly used in developed European and American countries, especially in patients with cardiogenic shock, right heart failure, or pulmonary hypertension (19), and since infarct size was considered a major prognostic factor, the limitation of myocardial size became a therapeutic pillar in the management of patients with myocardial infarction, and it was due to Chazov that thrombolytic therapy was introduced as a treatment for myocardial infarction (8).

## 3. Cardiac intensive care unit (CICU)

### 3.1. From coronary intensive care unit to cardiac intensive care unit

After validating the effectiveness of coronary intensive care units, and overcoming the main etiologies of mortality in patients with MI, it was observed that patients hospitalized in a CCU may require artificial ventilation, renal replacement therapy, central venous access, and cardiopulmonary arrest management, thus, cardiovascular emergencies are not only limited to the management of MI but also valvular disease, decompensated heart failure, severe pulmonary embolism, severe rhythm and conduction disorders, and postoperative cardiac surgery patients, for which coronary intensive care units evolved into what is now called cardiac intensive care units (CICUs) (20).

This concept of the CICU is quite recent (21), and it is only since the beginning of the 21st century that we started to talk about it. It is a unit that is responsible for providing increasingly complex care requiring a high level of skills to manage both cardiac and non-cardiac problems. This complexity is explained by several factors which include the age-dependent demographics of the population and associated comorbidities, the evolution of circulatory support modalities for refractory heart failure, the evolution of strategies after recovered cardiac arrest, and also the evolution of recommendations for the management of acute coronary syndromes.

It should be noted that today the ICU is no longer a cardiac rhythm monitoring unit, but rather a landing platform for patients with several associated comorbidities (27), on the one hand because of their overly charged surgical medical history, and on the other because of the complexity of the admitting pathology (22).

### 3.2. The definition of a CICU

The CICU is an administratively identified hospital unit, responsible for the specialized management of acute cardiovascular diseases. This unit is able to offer patients continuous telemetric monitoring and is thus characterized by the availability of medical and paramedical staff trained in the management of cardiovascular emergencies (23). This unit must have a well-defined organization in order to offer expertise 24 h a day, 7 days a week, in the management of acute cardiovascular diseases in consultation with the other specialties of the hospital.

Among the responsibilities of this unit is to provide a specialized cardiovascular environment to manage hospitalized patients in their entirety and not only on the cardiovascular level, as well as to ensure follow-up at discharge and in the long term. The CICU is responsible firstly for ensuring immediate access to care for clinically unstable patients by assisting with failing vital functions in patients with acute cardiovascular conditions, secondly for managing the admitting pathology, and then for ensuring a long-term specialized cardiovascular follow-up. For this, each ICU must have the appropriate equipment, technologies, and diagnostic means, as well as all the therapeutic means, whether medical, interventional, or surgical, in order to take care of the patient in accordance with the guidelines of the learned societies.

The medical responsibility for a ICU is assigned to a specialized cardiology team, under the supervision of a cardiology director, who decides on the care of all patients. Ideally, these medical staff should have qualified training in cardiac intensive care and ICU management (24).

### 3.3. Similarities and differences between an intensive care unit (ICU) and CICU

Given the current development in technology and increasingly sophisticated therapeutic means, there are now many similarities between ICUs and CICUs, but there are several important differences, mainly in the phenotype of patients admitted to ICUs compared to CICUs.

**Figure 3:**
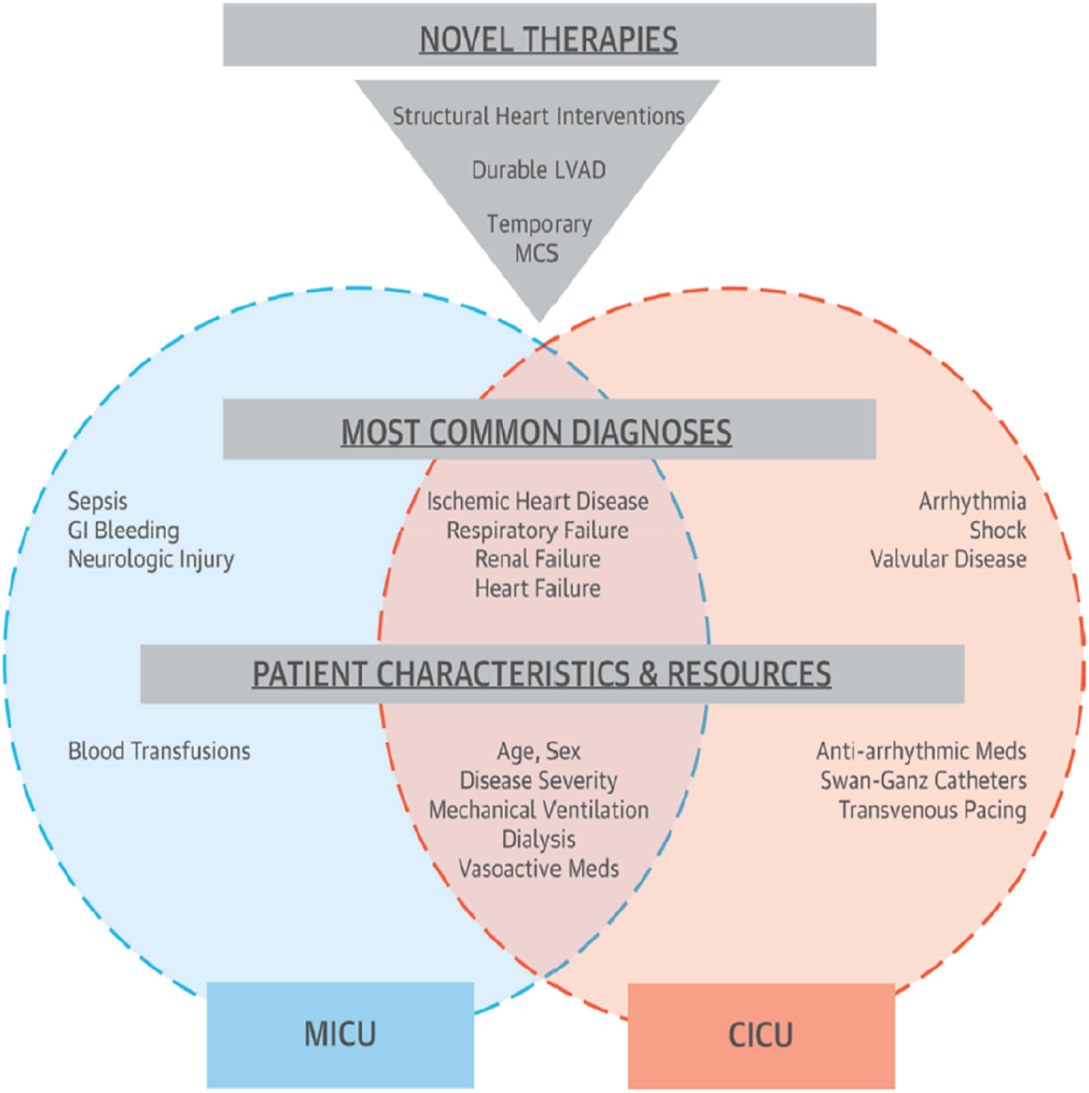
The possible differences and similarities between ICUs and CICUs (5).

If the pathology of admission in a CICU remains an acute cardiovascular condition, then this is not the case in an ICU, since the diagnosis of admission can be a severe trauma up to septic shock. Although the pathology of admission to a CICU is acute cardiovascular disease, the patient has every right to develop hemorrhage, respiratory failure, or infection for that matter, the ICU team must first be intensivist before being cardiologic, and if this is not the case, then there will be an inescapable collaboration between the ICU and CICU care teams (Figure 3) (25).

In order to properly manage these patients with cardiovascular conditions, but with respiratory, neurological, and renal repercussions, management protocols must be codified and written in collaboration with the ICU medical team.

### 3.4. The organizational model and human resources of a CICU

The organizational structure of an intensive care unit has always been a subject of debate since all studies conducted in this sense have confirmed that this organizational model is a determining element in the quality and short- and medium-term outcome of care (26).

Cardiac intensive care units are classically divided into closed and open units. In an open unit, several physicians can admit patients and decide on the therapeutic management, thus ensuring full medical responsibility for all patients, whereas, in a closed unit, the admission of patients is under the responsibility of a single physician, who directs the therapeutic decisions. This organizational concept is not only delimited by the decision to admit patients and the therapeutic decisions but also by the staffing. In the case of an open unit, the staff is not constant, and of different disciplines, but in a closed unit, the staff is the same for all patients, and of the same discipline. Another advantage to be added for closed units is the fact that having an administrative framework allows the adjustment of the vision between the different stakeholders in the management of the patient to have well-defined protocols and objectified progress.

In 2019, a systematic review with a meta-analysis was carried out by Qian Yang et al. (27) on the mortality and clinical course of patients hospitalized in a closed intensive care unit compared to an open intensive care unit and found a higher mortality rate in open vs. closed units with (OR: 1.31, 95% CI: 1.17–1.48; *p* = .00001). Another systematic review with meta-analysis was published in 2021 with more studies included (28). The result showed that the mortality rate in closed units was lower than in open units, but with no change in overall mortality, length of hospital stay, or severity of clinical characteristics. This difference could be explained by the constant presence of an intensivist in closed units, as well as the codification of protocols and therapeutic decisions in these closed units.

Among the reasons that explained this superiority of closed units over open ones was the satisfaction of the nursing staff in this type of unit, as well as the improvement and prioritization of responsibilities and communication among the nursing staff (29).

Regarding the difference between closed and open CICUs, few studies have been published in this sense. In 2021, a retrospective study over a period of four years was conducted in the United States to objectify the difference between demographic characteristics, clinical, management, and in-hospital mortality, at 30 days and after 1 year of a stay in a closed vs. an open CICU (26). Results for demographic parameters were similar. With regard to interventional procedures, closed ICUs performed more procedures than open ICUs. In terms of outcomes, ICU mortality rates were lower in closed units (6.9%) than in open units (7.3%) (OR, 0.70; 95% CI: 0.52–0.94; *p* = 0.02), and for median length of stay and in-hospital management costs, there was no difference between the two models. The same study was carried out by Katz et al. in Germany to compare the effectiveness of the two models on CICUs, the result did not show a significant difference in mortality but the length of hospital stay was reduced in the closed units compared to the open ones (30).

In conclusion, despite the difference that was limited to mortality for some studies, and to the length of hospitalization for other studies between the closed and open model, the superiority of the closed ICUs remains well established and clearly saw an improvement in communication, satisfaction, and thus therapeutic protocols compared to the open models.

The organizational model by itself is not enough to have optimal performance in a CICU, the human resources of a CICU represent an essential unit in the quality of care.

• The human resources of a CICU are represented as follows:
  1. The medical staff

The medical staff of an ICU is the medical team in charge of taking care of the patients in consultation with the paramedical team under the direction of a unit director. This staff is usually made up of cardiology residents in training with or without the presence of a cardiologist intensivist. The presence or absence of an intensivist from a CICU is an issue that has been the subject of much controversy (31).

A study by Na et al. discussed the association between mortality in a CICU and the presence or absence of an intensivist in the unit (32) and found very high mortality in the group where the intensivist was absent by a percentage of 8.8% compared to 4.1% with a statistically significant difference (*p* < 0. 001). Another study by NA et al. compared the survival of patients with cardiogenic shock between units with an intensivist and without an intensivist (33) and the result was surprising, with a mortality of 30.6% in units without intensivists with a mortality of 17.6% in units with an intensivist and a clearly significant difference (*p* < 0.001) between groups.

However, the phenotype of patients admitted to the CICU is not always stable (24) which leaves us to ask the question: when and for which patient phenotype is the presence of an intensivist in a CICU mandatory? This is the question that will be answered in the section titled “CICU classification”.

2. The nursing staff

The nursing staff has represented a pillar in the care of patients admitted to the CICU since the creation of the first unit (34), and despite the considerable advances in technology currently used in the CICU, the responsibility and importance of the nursing staff are constantly increasing. In recent years, attention has been focused on the adequate level of nurse staffing, and this is secondary to several studies that have confirmed that the system adopted for nurse staffing is closely related to the evolution of patients (5). Each healthcare system has proposed an optimal level of nurse staffing but without international consensus. In North America, there is a staffing system that is standard for all intensive care units. In the United Kingdom, recommendations are proposed but not mandated by law (34).

A concept has been proposed to find the optimal staffing of nurses, named the nurse-patient ratio (NPR). In general, the ratio used in the majority of CICUs is 2:1. In 2018, a systematic review with meta-analysis was performed to study the NPR by Driscoll et al. (35) but high heterogeneity was found in the method of measuring the NPR. The most used method was the calculation of the NPR by teams (25, 34). The result of this meta-analysis revealed that a higher level of paramedic staffing was associated with improved in-hospital survival, but without being able to define an optimal level required for the NPR. Among the parameters that demonstrate the crucial role of nurses is the impact of the level of nurse staffing on hospital evolution, mortality, and length of hospitalization. Several authors have been interested in objectifying the link between these parameters. Kim et al. studied the relationship between length of stay and NPR (36) and found a significant reduction in the length of hospitalization in centers with high nurse staffing, especially in centers that took care of critically ill patients. In 2020, Chang et al. published their work on the relationship between mortality and nursing staff, and they found that the lower the NPR, the higher the mortality, especially in patients with multiple comorbidities (37).

3. Clinical pharmacists

Pharmacy has undergone a spectacular evolution in recent years, moving from a fairly passive role as a supplier of medicines to a more active role by becoming involved in the management of patients alongside other healthcare providers such as doctors and nurses (38). Clinical pharmacists are health professionals specialized in therapeutics and are qualified to indicate global management of medicines to patients, physicians, and the rest of the health care team, whose main goal is to improve the quality of life, the efficiency of care, and thus the safety of patients (39). Currently, the role of the clinical pharmacist is well demonstrated in CICUs (40). A study by Xu et al. (41) showed a 66% reduction in adverse drug events in the same CICU after the integration of pharmacists into the visit with the ICU team, with a decrease from 10.4 events to 3.5 events per 1,000 patient days (*p* < 0.001).

At present, the clinical pharmacist represents an important actor in the process of treating patients with acute cardiac disease, following the introduction of the principle of multi-disciplinary management in all consensus and guideline documents issued by scientific societies. Taking heart failure as an example, the clinical pharmacist has an essential role to play in management, from initiation of treatment, titration and adjustment of doses, monitoring and reporting of adverse effects, possible interactions with other prescribed drugs, and long-term monitoring of the efficacy of prescribed drugs in collaboration with the treating physicians (42).

4. Nutritionists and dieticians

The role of nutritionists in an ICU is well known because the majority of patients hospitalized in these units have several cardiovascular risk factors (40) or are elderly, bedridden patients with severe malnutrition, for which nutritional management is essential in an ICU. A multicenter study carried out in European ICUs has clearly demonstrated the role of the presence of a nutritionist in a unit and its impact on intra- and extra-hospital evolution (43). Today, it has been demonstrated that malnutrition has a negative impact on the prognosis of patients admitted to cardiac intensive care units, whether in the short or long term. This effect is explained by the adverse impact of malnutrition on the immune system of these patients, making it fragile, and resulting in an increase in nosocomial infections. Other hypotheses that explain the negative impact of malnutrition include sarcopenia and accelerated catabolism of the organism, which are at the root of the inflammatory mechanisms of acute decompensation in chronic heart disease. This association of malnutrition and acute decompensation would have severe metabolic, hemodynamic, and neurological consequences (44).

Sugita et al. studied the correlation between nutritional status and delirium in 653 patients admitted to the coronary intensive care unit of Juntendo University Hospital. Nutritional status was assessed by three different scores: Geriatric Nutritional Risk Index (GNRI), Controlling Nutritional Status (CONUT) and Prognostic Nutritional Index (PNI). Results after multivariate analysis on several models showed that the PNI and CONUT were independent risk factors for the occurrence of delirium, demonstrating the seriousness of this neglected comorbidity (44).

5. Physiotherapists

The impact of chronic cardiovascular disease, mainly heart failure, on physical and musculoskeletal function has been widely demonstrated, making these patients, in addition to their multiple comorbidities and generally advanced age, more fragile, with a consequent reduction in autonomy and quality of life. For all these reasons, physical rehabilitation through physiotherapy has a significant role to play in the management of patients admitted to cardiac intensive care units (45). In a multi-center, randomized, attention-controlled trial to evaluate the value of early rehabilitation in 349 patients hospitalized for decompensated acute heart failure, the results showed a significant improvement in their quality of life at 3 months post-hospitalization, with improvements in the Short Physical Performance Battery (SPPB), 6-minute walk distance test, and the Kansas City Cardiomyopathy Questionnaire as well as a decrease in depression as assessed by the Geriatric Depression Survey-15 (46).

6. Other personnel

Other personnel are also necessary in the CICU such as medical assistants and radiology technicians.

### 3.5. The concept of a multi-disciplinary approach

The complexity and severe comorbidities of patients hospitalized in a CICU require intervention between the different specialists on the one hand and the different members of the integral care team in the CICU, namely, physicians, nurses, medical assistants, and clinical pharmacists, on the other. Several studies have demonstrated the effectiveness of the multidisciplinary approach in CICU patients.

Nutritionists, physical therapists, and social workers also play a major role in the management of patients with heart disease. Improved survival has been observed in units that have adopted this multidisciplinary management approach. In a Pennsylvanian CICU study, ICUs with “high-intensity” medical staffing had lower mortality than other ICUs (or 0.78, 95% CI 0.68–0.89; *p* < 0.001), and multivariate analysis showed that multidisciplinary care was associated with significantly reduced mortality (or 0.84, 95% CI 0.76–0.93; *p* ¼ 0.001).

Another major determinant in the multidisciplinary approach is communication. Clear communication among the increasing number of team members responsible for the management of critically ill patients is necessary for effective, high-quality care. A study at Johns Hopkins Hospital showed that increased communication using a daily goal form during ICU visits reduced the average length of stay in the intensive care unit by 50%, from 2.2 to 1.1 days (47).

### 3.6. The classification of the CICU

A three-level classification was proposed by the Association for Acute Cardiovascular Care of the European Society of Cardiology for the CICU (48). This classification can be made based on the phenotype of the patients or the type of technology and equipment available, the level of care presented, and finally the staffing.

***CICU level i***: refers to patients with acute cardiovascular conditions whose needs cannot be met by the care provided in a general cardiology department because their condition is likely to worsen and they require special expertise, specific equipment, or higher levels of monitoring.
***CICU level ii*:** level ii concerns patients with acute cardiovascular pathologies whose risk requires more thorough monitoring than level i.
***CICU level iii***: this level concerns all patients with acute cardiovascular pathology requiring acute circulatory assistance such as ECMO, invasive mechanical ventilation, or renal replacement therapy.

This classification can be made according to the following determining factors:

• Pathologies treated.
• Expertise and techniques.
• Equipment and technologies.
• Staffing and networks.

#### 3.6.1. Classification of admission pathologies according to CICU levels

The Association for Acute Cardiovascular Care proposed the following classification of the pathologies treated according to the level of CICU (Table 2).

**Table 2:** The classification of pathologies of admission in CICU according to the level of severity.

Level I pathology	Level II pathology	Level III pathology
Acute congestive heart failure	Acute heart failure with signs of hypoperfusion	Cardiogenic shock
Ventricular tachyarrhythmia without hemodynamic consequences	Acute heart failure with oligo-anuria	Cardiac arrest
Uncomplicated stemi after revascularization	Need for vasopressors (shock, sepsis…)	Hemodynamically poorly tolerated ventricular fibrillation or ventricular tachycardia
Uncomplicated high risk ischemic nstemi	Arrhythmia complicated by heart failure	Mechanical complications of ACS
Acute pulmonary edema	Non-revascularized stemi or nstemi at high or very high ischemic risk	Heart transplant recipient with suspected graft rejection
Atrial fibrillation complicated by heart failure	Stemi or nstemi complicated by heart failure without shock	Infectious endocarditis with heart failure
Uncomplicated myopericarditis	Complication of coronary angiography or PCI	Aortic regurgitation with heart failure
Uncomplicated pulmonary embolism	Acute mitral regurgitation with heart failure	Thrombosis of a valve prosthesis
Non fulminant myocarditis	Severe aortic stenosis with signs of heart failure	Aortic dissection type a
Peripartum cardiomyopathy	Cardiac tamponade	Uncomplicated type B aortic dissection
Complicated or uncomplicated mitral stenosis	High intermediate risk pulmonary embolism	Any level II situation in aggravation
	Uncomplicated type b aortic dissection	Massive pulmonary embolism
	Peripartum myocarditis or cardiomyopathy with reduced left ventricular ejection fraction	

#### 3.6.2. Classifications of equipment and technologies according to CICU levels

##### 3.6.2.1. Classification of techniques and expertise according to CICU levels

For techniques and expertise, the Association for Acute Cardiovascular Care proposed a classification according to the techniques and expertise available in the CICU (Tables 3, 4) and those available in the hospital facility to which the CICU belongs (Table 5).

**Table 3:** The equipment and technologies required according to the level of the CICU.

CICU level I	CICU level II	CICU level III
At least two ECG machines	All the equipment and technologies offered in level I	All the equipment and technologies offered in level II
Non-invasive blood pressure monitor	An extra ECG machine	Advanced invasive hemodynamic monitoring
At least one monitor for invasive blood pressure monitoring	Invasive blood pressure monitor	Right catheterization equipment
Pulse oximetry	Capnography equipment	Hemodialysis and hemofiltration equipment available in CICU
Electronic medical records archiving system with electronic prescription system	Invasive hemodynamic monitoring	Device for maintaining therapeutic hypothermia available in CICU
Telemetric monitoring of all patients	Respirator for mechanical ventilation	Circulatory assistance such as ECMO and IMPELLA
Electrical patient monitoring stations for nurses	Mobile echocardiograph with a trans-esophageal sonde	
A syringe pump	An aortic counter-pulse balloon	
Positive pressure ventilation system (CPAP)	Hemodialysis and hemofiltration equipment available in the hospital facility	
A biphasic defibrillator	Device for maintaining therapeutic hypothermia available in the hospital facility	
A ventilator for non-invasive ventilation		
Mobile echocardiography		
An electro-systolic temporary pacing probe		
Blood gas analyzer		

**Table 4:** Techniques and expertise needed in the CICUs.

CICU level I	CICU level II	CICU level III
Non-invasive monitoring of all clinical parameters	Placement of central venous accesses	Setting up and managing ECMO-type circulatory assistance
24/7 availability of an echocardiologist	The realization of pericardial drainage	The initiation and management of renal replacement therapy
Electrical cardioversion available 24/7	Performance of trans-esophageal echocardiography	The management of a mechanical ventilation
Non-invasive ventilation	Performing a pulmonary artery catheterization	
Temporary cardiac pacing available 24/7	Performance of a circulatory assistance such as aortic counterpulsation balloon	
Nutritionist team available in CICU	Thermal management of patients	
Physiotherapy and physical rehabilitation team available in CICU		

**Table 5:** Techniques and expertise required in the hospital structure to which the CICU.

CICU level I	CICU level II	CICU level III
A functional emergency department	A 24/7 functional coronary catheterization laboratory	A cardiovascular surgery team with expertise in coronary surgery, aortic surgery, valve surgery and all structural pathologies of the heart
A 24/7 functional radiology department for standard radiology, CT scan	A cardiac pacing and resynchronization program available	An interventional radiology department with expertise in the endovascular treatment of aortic diseases
The availability of an echocardiography device with trans-esophageal probe	A pacemaker and defibrillator implantation program available	An interventional radiology service available with expertise in arterial embolization
The availability of a palliative medicine service	A cardiac ablation program available	An interventional radiology department available with expertise in vascular neuro-radiology
A 24/7 functional biology laboratory for cardiac enzymes	A functional nephrology department	The availability of an interventional cardiology team with expertise in the treatment of valvulopathy by percutaneous means
A 24/7 functional biology laboratory for haemostasis tests	Magnetic resonance imaging available	Availability of a functional heart transplant program
A 24/7 functional biology laboratory for renal and hepatic assessment	A team trained in post-cardiac arrest care	
	Availability of a team trained in endo-myocardial biopsy	

##### 3.6.2.2. Classification of staffing and network according to CICU levels

CICU *level i:*

• The management of these units is given to a cardiologist.
• Expertise in 24-h echocardiography is required.
• The recommended nurse-patient ratio is one nurse for four patients.
• This level of CICU must be in close contact with the different disciplines of the hospital and thus constitutes the first line of care for acute cardiovascular diseases.

CICU *level ii:*

• The management of a level ii CICU must be performed by an intensive care cardiologist.
• The nurse-patient ratio for this level is: one nurse for every two patients with a maximum of one nurse for every three patients.
• In these guidelines, ESC proposes the following formula, but it is a formula that remains to be discussed: four beds in CICU for every 100,000 inhabitants.

CICU *level iii:*

• The director of the unit must be a cardiac intensivist with proven experience and competence in acute cardiovascular care.
• The nurse-patient ratio must be one nurse for one patient and at most one nurse for two patients.
• The presence of an interventional cardiologist, a cardiac surgeon, and an anesthetist is necessary in the unit.

## 4. Performance indicators for a CICU

If the 21st century has seen a revolution in the development, standardization, and normalization of care in acute cardiovascular medicine, through the formalized recommendations of experts from learned societies, then there is still a wide divergence in current practice with the aim of reducing the difference between the care performed and the evidence-based care, thus to standardize and prioritize the management of the different patients in a CICU, with an objective evaluation of the effectiveness and performance of the latter, quality or performance indicators are proposed and increasingly used by the different directors of modern CICU (49).

**Figure 4:**
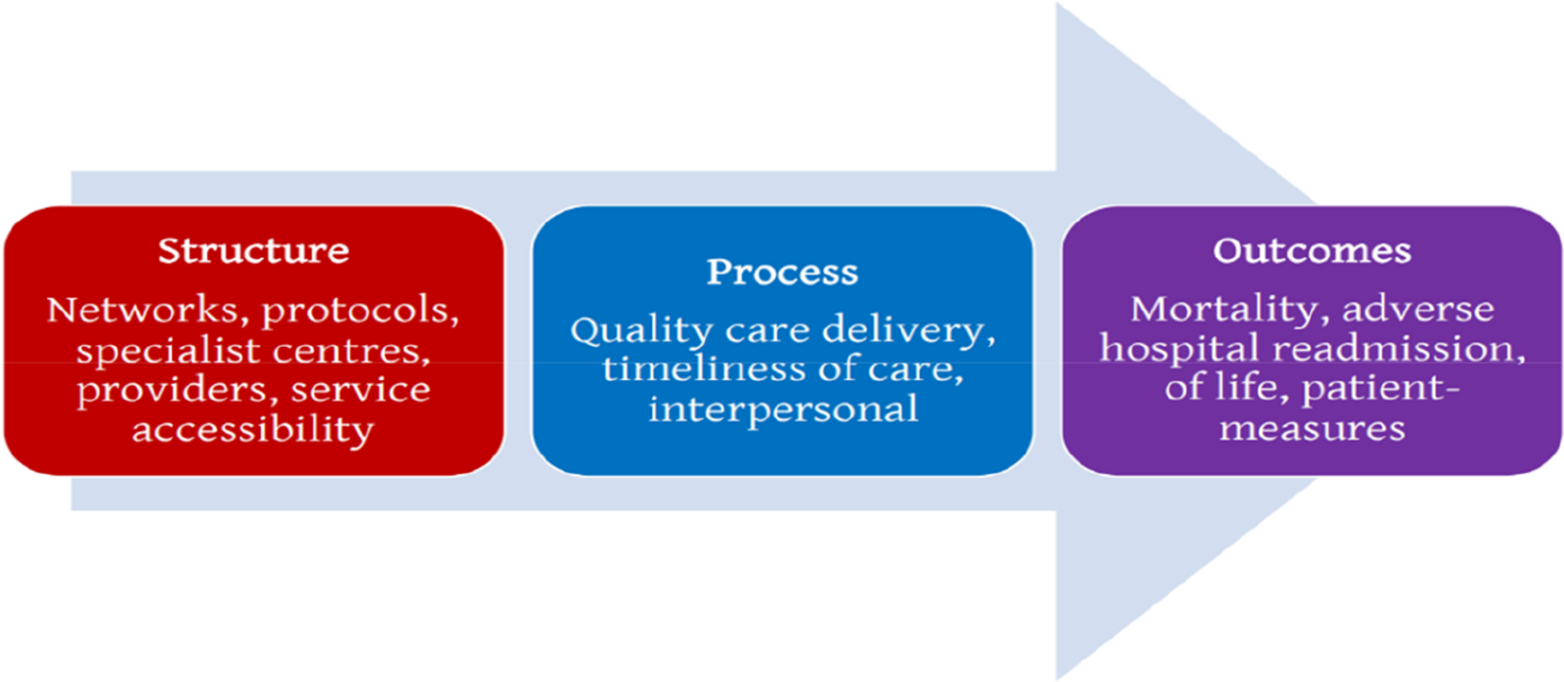
Diagram proposed by the European society of cardiology to classify the different performance indicators.

The European Society of Cardiology has divided quality and performance indicators into three types (Figure 4) (50).

• **Structure indicators**: these describe the structural organization, staffing, technologies, and equipment available.
• **Process indicators**: these describe the therapeutic protocols used, as well as compliance with the guidelines of learned societies.
• **Outcome indicators**: these describe the intra- and extra-hospital evolution of patients, in terms of mortality, length of stay, readmission rates, and the patient's perception of the care provided.

**Figure 5:**
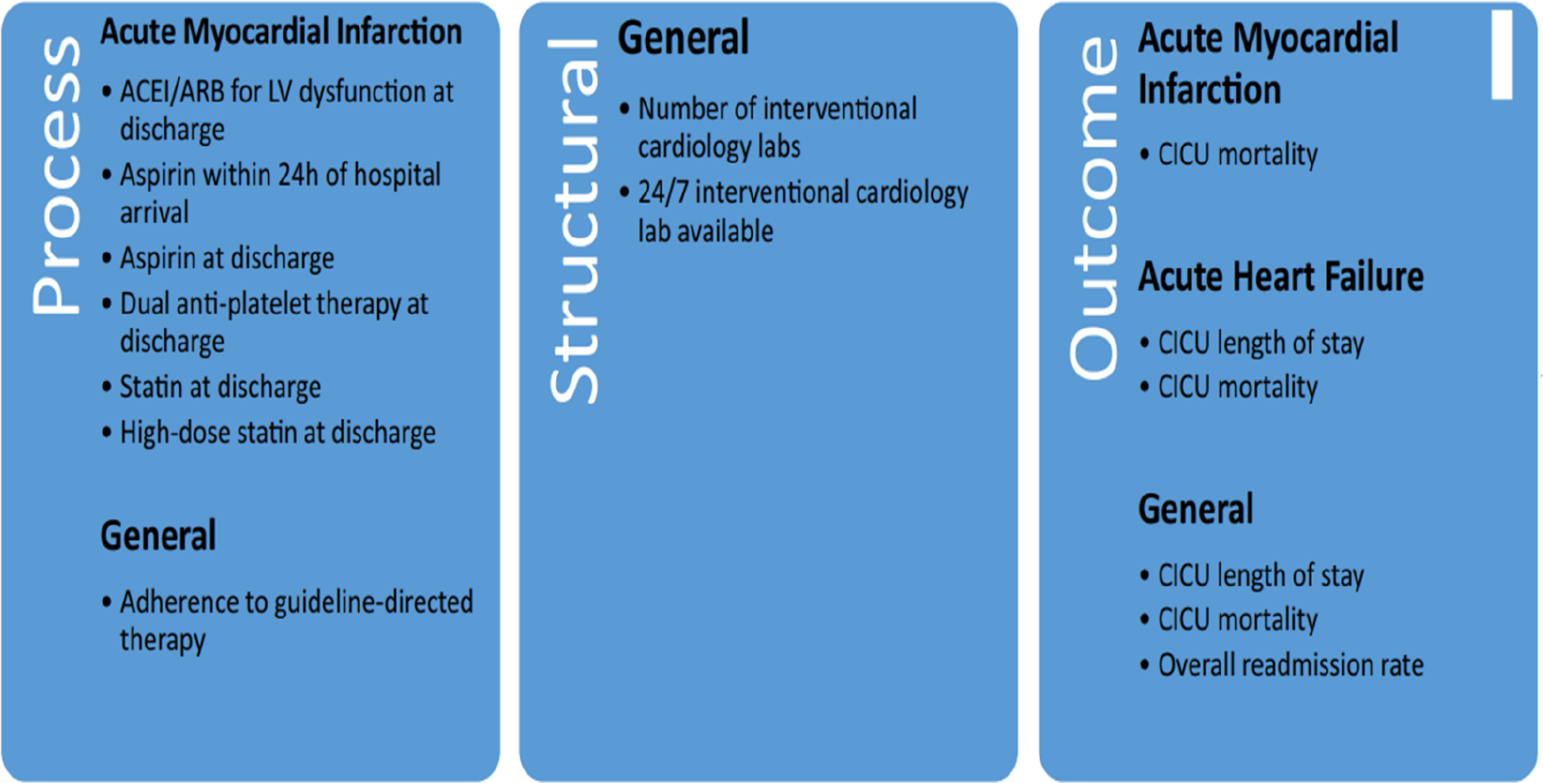
The different indicators of quality and performance of a CICU proposed by Goldfarb et al.

In 2019, Goldfarb et al. (51) (Figure 5) conducted a systematic review with the main objective of determining indicators of the general performance of a CICU apart from specific indicators for a specific pathology and the results are as follows:

Among the 108 quality indicators found:

• 70 were proposed as process indicators.
• 19 were proposed as structure indicators.
• 19 were proposed as indicators of results and evolution.

To date, there are no well-established recommendations for assessing the functionality of a CICU beyond the previously cited classification proposed by the Association for Acute Cardiovascular Care, but the results of this systematic review remain applicable.

## 5. The training program in a CICU

In 2020, seeing the increasing demands on the practice of cardiology as well as the increased training needs, the European Society of Cardiology together with the European Union of Medical Specialists, have worked on a core curriculum for cardiologists that has been published in order to bring the visions together (52).

### 5.1. Objectives of intensive cardiology training

Cardiology patients remain a very special subtype of patients since they can be treated in ambulatory care, as well as hospitalized in a cardiological intensive care unit. For this reason, a cardiologist must have both professional skills for the management of stable patients without compromised vitals and for the management of unstable patients with vital prognoses in danger.

For this purpose, five objectives have been specified by the ESC for the training program of cardiologists with regard to intensive cardiology (53):

(1) Management of a hemodynamically unstable patient.
(2) Management of a surviving cardiac arrest patient.
(3) The management of a critically ill cardiac patient.
(4) The management of a patient after an interventional cardiology procedure.
(5) Management of a cardiac patient requiring end-of-life care.

### 5.2. Levels of independence in intensive cardiology

The ESC classifies the levels of independence expected of a cardiology trainee into five levels (54):

• Level 1: the trainee should only observe.
• Level 2: the trainee should be able to perform an activity but under direct supervision.
• Level 3: the trainee should be able to perform an activity but under indirect supervision.
• Level 4: the trainee must be able to perform an activity but with remote supervision (the supervisor must be available in less than 20–30 min).
• Level 5: the trainee must be able to supervise other trainees.

## 6. Research in CICUs

The current evolution of intensive cardiology represents a real focus for new studies and research. Given the spectacular progress of medical technology and its integration into the care of patients, especially those in the ICUs, several research topics are currently posed, especially with regard to mechanical circulatory assistance devices and thus the study of myocardial dysfunction during sepsis (55). The results of this research will undoubtedly contribute to an improvement in patient care, and thus to the standardization and creation of well-defined and more efficient functional ICU models.

The key elements to initiate and develop research topics in CICUs are:

(1) the creation of computerized databases for efficient data management.
(2) the organization of research teams.
(3) creation of multi-center and internationally focused research networks.
(4) getting support from academic organizations, government agencies, etc.
(5) ethics in a CICU.

The serious and unstable nature of CICU patients makes the ethical aspect somewhat complex, as neither the patients nor their relatives can often participate in the decision-making process regarding care. Considering that the main clinical characteristic of patients hospitalized in a CICU was a poor vital prognosis, the care team of a CICU must be well prepared and wise in the presence of a death, with all the possible ethical aspects.

Some of the ethical challenges in a CICU include writing a discontinuation of care form, negotiating with family members not to inform the patient of their diagnosis or vital prognosis, answering an interesting question, the prognosis of a patient with end-stage cardiovascular disease, and making the decision about end-of-life care.

One of the major determinants of ethical aspects in the CICU is the economic challenges and thus the limited resources, for example, in the United States, a bed in a CICU costs between 4,000 and 10,000 dollars per day (56). For this reason, prolonged care for patients with poor prognosis in the CICU is a great subject of debate, but the decision to limit care for critically ill patients for reasons of limiting economic expenses remains a real ethical challenge.

### 6.1. Practical guidelines for ethical decision-making

In order to make an ethical decision, the following four steps are recommended:

(i) Consider patients as major stakeholders in healthcare decision-making.
(ii) Define the person who has the authority to make the medical decision.
(iii) Communication.
(iv) Determination of patients' values.

This fourth point also remains difficult to determine and consists of the extent to which a painful experience is accepted by the patient. This question can only be answered by the patient, and may vary in terms of prognosis and how the patient advocates the definition of quality of life and thus their power to cope with the difficulties of care and the indignities of the disease, both moral and financial.

### 6.2. Discontinuation of care and end of life in the CICU

Discontinuation of care is the most difficult action a clinician can take. If the role of the physician is to care for patients, improving their prognosis and thus quality of life, for seriously ill patients with a therapeutic impasse, the best solution may be to propose end-of-life care for a death that is as dignified and pain-free as possible (57). In some cases, offering end-of-life care for a relatively painless and dignified death remains the best decision the healthcare team can make (58).

## 7. The perspectives and challenges in CICUs

Cardiovascular intensive medicine is constantly evolving, and despite all the current advances in recommendations for the organization, staffing, therapeutic management, and classification of the ICUs, as well as the magical evolution of technology and medical equipment many challenges and challenges are to be faced in the future in CICUs (59). In this section, we will try to mention the main challenges of modern CICUs:

### 7.1. Patient management after complex interventional procedures

**Figure 6:**
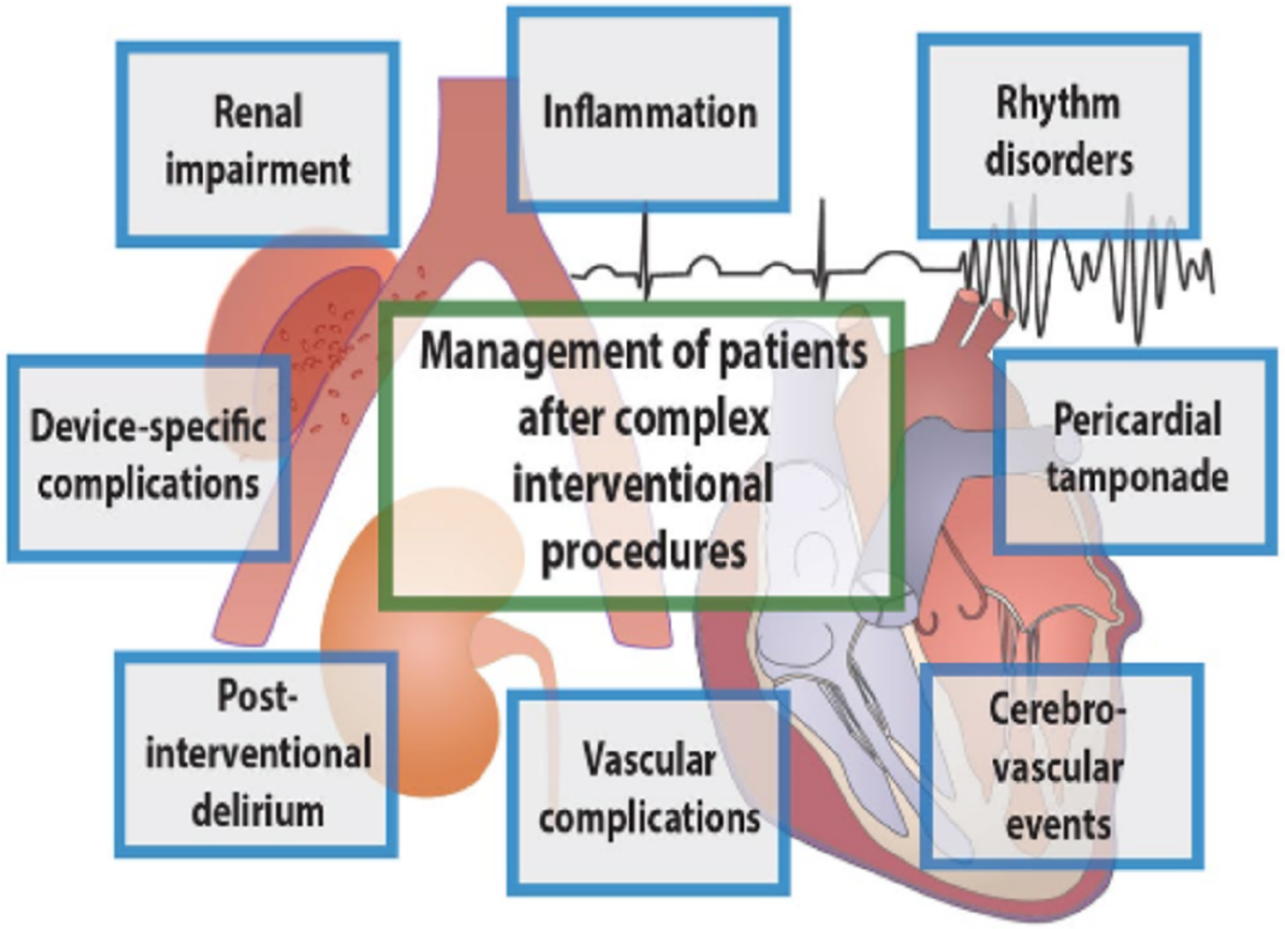
The various complications to be managed in complex post-interventional procedures [by Lüsebrink et al. (1)].

Given the high frequency of complex interventional procedures in CICUs, such as percutaneous aortic valve replacement (TAVI) (60), percutaneous mitral valve repair (mitral-clip) (61), percutaneous left atrial closure (62), and percutaneous dilatation of chronic coronary occlusion (CTO) (63) as well as the high complication rate after these procedures, the CICU staff must have continuous and updated training in order to be able to decrease the morbi-mortality rate after these procedures. The main complications to be managed in these patients are as follows (Figure 6):

(1) vascular complications.
(2) cerebrovascular events.
(3) cardiac tamponade.
(4) arrhythmias and cardiac conductance disorders.
(5) post-interventional delirium.
(6) renal dysfunction.
(7) inflammation.

Management of cardiogenic shock and the concept of a “shock team”.

Cardiogenic shock is always a subject of debate for cardiac intensivists because, on the one hand, of the problem of definition that poses it, and on the other hand, the difficulty of therapeutic management, given that the majority of guidelines mainly focus on the management of cardiogenic shock of ischemic origin as the most frequent cause of this condition (64).

Among the concepts currently adopted by several CICUs to improve the management of cardiogenic shock and its prognosis, is the shock team concept, which consists of a multidisciplinary management between interventional cardiologist, cardiologist, cardiac surgeon, and cardiologist intensivist. This approach has proven its effectiveness, especially in terms of a good individualization of the phenotypes of the patients through the more frequent use of invasive hemodynamic monitoring and catheterization of the pulmonary artery. This allows a more relevant use of circulatory support with a more adequate timing (65).

### 7.2. Management of post-cardiac arrest and maintenance of targeted temperature

The management of cardiac arrest and especially its post-recovery resuscitation remains a real challenge for all intensive care units (66). The CICU represents one of the basic units for the specialized management of cardiac arrest. For this, the staff of these units must be able to manage both the resuscitation of cardiac arrest and post-cardiac arrest resuscitation (67) and to achieve this result, continuous training, as well as an updating of knowledge, is necessary in order to improve the morbi-mortality of this pathology (68, 69).

### 7.3. Management of patients undergoing circulatory assistance and its complications

The use of circulatory assistance in CICUs has increased exponentially, especially after the modernization of the majority of ICUs in European countries (70). This use requires a heavy technical platform, with well-trained medical and paramedical personnel with the capacity to manage both the patient and the assistance, and also the complications of this circuit, which represent the principal cause of mortality in these patients (71). For all these reasons, modern ICUs must offer continuous training programs for all personnel on the management of patients on life support and thus determine well-defined protocols for the management of complications based on international guidelines.

### 7.4. Artificial intelligence (AI) in CICUs

The complexity as well as the severity of the patients admitted in CICUs makes this population quite special and requires personalized management based on several parameters mainly clinical, electrocardiographic, biological, and echocardiographic, in order to stratify the severity of these patients to predict the prognosis. With the evolution of artificial intelligence, it has been shown that several automated and dynamically evaluated algorithms can predict the evolution during hospitalization in CICU in a pertinent way (72).

Since 2020, several algorithms have been developed for the prediction of mortality or left systolic dysfunction in patients with atrial fibrillation or for patients hospitalized in the CICU, using ECG-based algorithms (73). The advantages include, in comparison with the scores developed in the past, the dynamic nature of the evaluation, and the fact that the gaps in the scores used are filled.

## 8. Conclusion

Cardiovascular diseases remain the first cause of mortality in all countries of the world whatever the level of development of the country, and the environments of cardiac intensive care units are clearly progressing with regard to their organization, management, and staffing; the introduction of the concept of indicators of the quality; and, with the objective of decreasing the rate of mortality, the cost of caring for these patients, which represents a real burden on the various healthcare systems.
